# Impact of simulated brain interstitial fluid flow on the chemokine CXCL12 release from an alginate-based hydrogel in a new 3D *in vitro* model

**DOI:** 10.3389/fddev.2023.1227776

**Published:** 2023-07-24

**Authors:** Wiam El Kheir, Anaïs Dumais, Maude Beaudoin, Bernard Marcos, Nick Virgilio, Benoit Paquette, Nathalie Faucheux, Marc-Antoine Lauzon

**Affiliations:** ^1^ Advanced Dynamic Cell Culture Systems Laboratory, Department of Chemical and Biotechnological Engineering, Faculty of Engineering, Université de Sherbrooke, Sherbrooke, QC, Canada; ^2^ Laboratory of Cell-Biomaterial Biohybrid Systems, Department of Chemical and Biotechnological Engineering, Faculty of Engineering, Université de Sherbrooke, Sherbrooke, QC, Canada; ^3^ Department of Chemical Engineering and Biotechnological Engineering, Faculty of Engineering, Université de Sherbrooke, Sherbrooke, QC, Canada; ^4^ Department of Chemical Engineering, Polytechnique Montréal, Montréal, QC, Canada; ^5^ Department of Nuclear Medicine and Radiobiology, Faculty of Medicine and Health Sciences, Université de Sherbrooke, Sherbrooke, QC, Canada; ^6^ Clinical Research Center of the Centre Hospitalier Universitaire de l’Université de Sherbrooke, Sherbrooke, QC, Canada; ^7^ Research Center on Aging, Sherbrooke, QC, Canada

**Keywords:** glioblastoma multiforme, nanoparticles, chemokines gradient, kinetic release, mathematical modeling, 3D delivery system, dynamic culture conditions, perfusion bioreactor

## Abstract

**Introduction:** Extensive investigation has been undertaken regarding drug delivery systems for the management of glioblastoma multiforme (GBM). The infiltrative behavior of GBM cells within the brain tissue is primarily attributed to their heterogeneity, the movement of interstitial fluid (IFF), and the presence of chemokines. These factors contribute to the limited effectiveness of current conventional treatments. To address the dissemination of GBM cells, a proposed therapeutic approach involves utilizing a controlled release gradient of CXC-chemokine-ligand-12 (CXCL12). However, the impact of IFF on GBM cell migration within the brain underscores its critical importance as a significant parameter, which, surprisingly, has not been extensively studied in the context of localized drug delivery targeting the brain.

**Methods:** Hydrogels are known for their inherent capacity to entrap various agents and exert precise control over their subsequent release. In the present investigation, we aimed to elucidate the release kinetics of CXCL12, whether in its free form or encapsulated within nanoparticles, from alginate-based hydrogels, both under static and dynamic conditions. To investigate the impact of convective forces mimicking the interstitial fluid flow (IFF) within the peritumoral environment of the brain, a three-dimensional *in vitro* model was developed. This model enabled the evaluation of CXCL12 release as a function of time and position, specifically accounting for the contribution of simulated IFF on the release behavior.

**Results:** We first demonstrated that the release kinetic profiles under static culture conditions were independent of the initial mass loading and the predominant phenomenon occurring was diffusion. Subsequently, we investigated the release of CXCL12, which was loaded into Alginate/Chitosan-Nanoparticles (Alg/Chit-NPs) and embedded within an alginate hydrogel matrix. Mathematical modeling results also indicated the presence of electrostatic interactions between alginate and CXCL12. The Alg/Chit-NPs effectively slowed down the initial burst release, leading to a reduction in the diffusion coefficient of CXCL12. To further study the release behavior, we developed a perfusion bioreactor with a unique culture chamber designed to recapitulate the peritumoral environment and varied the fluid flow rates at 0.5 µL/min, 3 µL/min, 6.5 µL/min, and 10 µL/min. As the flow rate increased, the cumulative amount of released CXCL12 also increased for all three initial mass loadings. Beyond 3 µL/min, convection became the dominant mechanism governing CXCL12 release, whereas below this threshold, diffusion played a more prominent role.

**Conclusion:** The indirect perfusion flow had a crucial impact on CXCL12 release and distribution inside the hydrogel in and against its direction. This system highlights the importance of considering the IFF in brain targeting delivery system and will be used in the future to study GBM cell behaviors in response to CXCL12 gradient.

## 1 Introduction

Glioblastoma multiforme (GBM) accounts for more than 82% of malignant gliomas and is considered the most aggressive and invasive brain cancer, with a worldwide incidence rate less than 10 per 100,000 (Dolecek et al., 2012; Ostrom et al., 2018). GBM is associated with the worst prognosis, with a survival rate of less than 5% at 5 years post-diagnosis (Dolecek et al., 2012). Indeed, GBMs are extremely complicated to eradicate by standard treatments due to their ability to infiltrate the brain and their resistance to chemotherapy and radiotherapy (El Kheir et al., 2022). Therefore, understanding the mechanisms responsible for GBM cell dissemination and the molecules involved in such phenomena is critical for developing new therapeutic approaches to treat this cancer.

It has been proposed that chemokines such as CXC-chemokine-ligand-12 (CXCL12), also known as stromal-cell derived factor 1 (SDF-1), be used to reverse the migration of GBM cells invading the brain parenchyma to trap them in an alginate hydrogel (Molina-Peña et al., 2021; Solano et al., 2021; El Kheir et al., 2022; Safi et al., 2022). Various studies have demonstrated that CXCL12, via its receptor C-X-C chemokine receptor type 4 (CXCR4), controls GBM migration (Do Carmo et al., 2010; Munson et al., 2013; Kingsmore et al., 2016; Cornelison et al., 2018). However, like other proteins, CXCL12 has a short half-life and must be loaded into hydrogels and nanocarriers such as nanoparticles (NPs). Considered highly biocompatible materials, hydrogels and NPs provide a series of advantages for delivery applications, such as drug transport across the blood–brain barrier and a sustained release to enhance their therapeutic efficiency. Hydrogels are 3D *in vitro* models permitted for the study of cancer cells in an *in vitro* environment that mimic, as closely as possible, *in vivo* conditions (Nii et al., 2020). Components of the cancer environment, such as the extracellular matrix (ECM), constitute an important factor in GBM progression (El Kheir et al., 2022). Therefore, natural biomaterials such as chitosan, alginate, hyaluronic acid, and Matrigel are often used to prepare and design 3D culture models (Nii et al., 2020). Usually, materials such as Matrigel and alginate or chitosan and alginate are combined (Nii et al., 2020). We have previously developed and characterized a composite NP delivery system composed of alginate and chitosan to entrap CXCL12 (Gascon et al., 2020). We determined an encapsulation percentage higher than 96.7% for a mass loading range of 0.37–1.49 µg of CXCL12/mg of NPs (Gascon et al., 2020). Different techniques are used to assess and quantify drug release from hydrogels (Vigata et al., 2020). Generally, in the context of a release kinetics study, drugs are released into a culture medium that mimics physiological conditions and supernatants are collected at several time points for drug concentration analysis and quantification (Vigata et al., 2020). Thereafter, a cumulative release curve is drawn which corresponds to the amount of the drug released over time (Vigata et al., 2020). From our previous study, release kinetic experiments revealed a burst release of CXCL12 in the first hours followed by a sustained release until a pseudo-plateau was reached, indicative of the presence of interactions between the drug and the delivery vehicle. Thereafter, we proved that alginate/chitosan nanoparticles (Alg/Chit-NPs) unloaded and loaded with CXCL12 are not toxic and promote GBM F98 cell migration (Gascon et al., 2020). The results highlight that the NPs do not affect F98 cell viability and proliferation (Gascon et al., 2020). In addition, the CXCL12 released from Alg/Chit-NPs significantly increased the migration of F98 cells through the layer of Matrigel at a concentration of 100 ng/mL (Gascon et al., 2020). Khan et al. (2023) demonstrated that GBM stem cells migrate in response to a CXCL12 concentration gradient ranging from 0.02 to 0.2 μg/mL. It was also demonstrated that CXCL12 stimulates SW480 and HT-29 metastatic colorectal cell line migration via its receptor CXCR4 at a specific concentration of 100 ng/mL (Rubie et al., 2011).

These experiments were performed in static conditions; however, there are a variety of phenomena that occur in the brain that affect the transport of molecules, particularly the interstitial fluid flow.

Interstitial fluid flow (IFF) is the movement of fluid between cells in the interstitial space and is mainly composed of water, ions, gaseous and organic molecules such as O_2_ and CO_2_, and hormones (Munson, 2019). IFF has been shown to alter cancer cell invasion, proliferation, and differentiation in primary brain tumors and metastases (Kumar and Weaver, 2009; Yu et al., 2011). Furthermore, it plays a critical role in the GBM cell invasion of the brain parenchyma by inducing an overexpression of CXCR4 and an increase in CXCL12 secretion along white matter tracts, blood vessels, and subpial regions (Zagzag et al., 2006; Munson et al., 2013). Magnetic resonance imaging (MRI) and intravital microscopy are the techniques commonly used to measure cerebrospinal, blood, and IFF dynamics. Depending on the region of the brain, IFF was found to be 0.61 mm/h in the white matter tracts of the temporal lobe of rats (Geer and Grossman, 1997), whereas bulk flow rates of 0.1–0.3 μL/min were reported in rat brains along the perivascular spaces and axon tracts (Abbott, 2004). In addition, it has been shown using MRI that the mean cerebrospinal fluid flow in junction between the aqueduct of Sylvius and the fourth ventricle was 3.44 mL/min and was 4.41 mL/min in the fourth ventricle in the human brain (Zhu et al., 2006). IFF was measured by Chary and Jain (1989) in normal and neoplastic rabbit ear chambers and was found to be 0.6 × 10^−4^ cm/s.

Kingsmore et al. (2016) used a three-dimensional (3D) *in vitro* interstitial flow model to find that the migration of different GBM stem cell lines increases in response to IFF through CXCR4/CXCL12 chemotaxis compared with static conditions. Their results confirmed the crucial role played by CXCL12 for flow-responsive invasion (Kingsmore et al., 2016). In that context, 3D *in vitro* models emerged as a better way to understand the physiological human context, and allowed the study of GBM migration, invasion, and cell–cell interactions while allowing them to express their aggressive and metastatic potential (El Kheir et al., 2022). For example, it has been demonstrated using a perfusion bioreactor that cancer cells are capable of migrating through a 3D *in vitro* dynamic culture model that mimics the peritumoral space. It also allowed cells to grow *in vitro* while expressing their *in vivo* properties such as invadopodia and migration capability (Cavo et al., 2018). However, while several 3D models have been developed to emulate cell behavior under dynamic conditions, it seems that no system focusing on the convective contribution of the IFF on drug release has thus far been reported in the literature. The use of a perfusion bioreactor system offers several advantages, such as precise control of the flow under physiological-like conditions. GBM tumors can be found in different brain regions, some more irrigated than others with fluids such as cerebrospinal fluid, blood, and interstitial fluid. This implies the presence of different IFF rates. Therefore, variation in the perfusion bioreactor flow rate might help guide future 3D drug delivery system design to allow a better understanding of fluid flow dynamic effects.

In the present study, we characterized the kinetic releases of CXCL12 at various initial mass loadings from different hydrogels, aiming to recapitulate the brain peritumoral environment under two release conditions: static and dynamic. Under static conditions, the release kinetics of free CXCL12 or CXCL12 encapsulated into Alg/Chit-NPs were analyzed from an alginate hydrogel 1% (w/v), and an alginate:Matrigel (Alg:M, 50:50) hydrogel. The release kinetics was mathematically modeled to estimate the effective diffusion coefficient and mass transfer coefficient at the surface. We then developed an *in vitro* 3D model that considers the convective contribution of the cerebral fluid flow to study CXCL12 release under conditions that mimic the human brain environment and IFF. Four flow rates which could enhance CXCL12 release *in vivo* depending on the tumor location were used. Finally, we analyzed the different sections of the 3D model to quantify the remaining trapped CXCL12 to get a better understanding of molecule distribution. To the best of our knowledge, this study represents the first utilization of such a system to elucidate the influence of flow dynamics within an *in vitro* 3D environment on the release of chemokines in the specific context of brain cancer.

## 2 Material and methods

### 2.1 Material

AlexaFluor^®^ 647 conjugated-human recombinant CXCL12α (CXCL12-AF647) was purchased from Almac Group (Craigavon, United Kingdom) and reconstituted in sterile Milli-Q water following the manufacturer’s instruction. Human recombinant CXCL12 (SDF-1α) (purity ≥98%, PeproTech United States, Rocky Hill, NJ, United States) was reconstituted in sterile Milli-Q water. PRONOVA^®^ UP LVG medical grade sodium alginate with a guluronic acid (G) to mannuronic acid (M) ratio ≥1.5 and apparent viscosity of 20–200 mPa∙s was purchased from NovaMatrix (Sandvika, Norway). High molecular weight (HMW) chitosan (>310 kDa) with deacetylation 85% and calcium chloride (CaCl_2_) was purchased from Millipore-Sigma (Sigma-Aldrich, Oakville, ON, Canada). Matrigel^®^ growth factor reduced (GFR) basement membrane matrix was purchased from Corning^®^ and thawed overnight at 4°C. Polydimethylsiloxane PDMS (SYLGARD™ 184 Silicone Elastomer Kit) was purchased from Ellsworth Adhesive Canada (Stoney Creek, ON, Canada). Antibiotic (100X) was purchased from Gibco (ThermoFisher, Burlington, ON, Canada), and cell culture medium DMEM 1X Multicell was acquired from Wisent Bioproducts (Wisent Inc, St-Bruno, QC, Canada).

### 2.2 Methods

#### 2.2.1 Nanoparticle synthesis, morphology, and distribution characterization

##### 2.2.1.1 Nanoparticle synthesis

Alg/Chit-NPs were synthesized as previously described (Gascon et al., 2020). Briefly, CXCL12 with or without AF647 conjugate was added to the alginate (1 mg/mL) to reach a final concentration of 1.6 μg/mL and left 5 min to agitate. Then, using ionotropic gelation and under aseptic conditions, a calcium chloride solution (CaCl_2_, 18 mM) was added dropwise to the solution of alginate under vigorous stirring. HMW chitosan solution (1 mg/mL in 1% (v/v) glacial acetic acid) was added dropwise 30 min after CaCl_2_ for a final ratio of 4:1, and the solution was left overnight under agitation at 4°C. The NP solution (10 mg/mL) was then centrifuged 20,000 × g for 30 min at 10°C. NPs were used to obtain three CXCL12 doses: 400, 800, and 1,600 ng/mL.

##### 2.2.1.2 NP zeta potential measurement

Alg/Chit-NPs were prepared with or without CXCL12 (10 mg/mL). The zeta potential of these Ag/Chit-NPs was characterized (NanoBrook Omni). Using 4 mL disposable polystyrene (aqueous) cuvettes, the samples were diluted in sterilized Milli-Q water to reach a final concentration of 25 μL/mL. The particles were not filtered prior to the measurements, which were performed at 25°C after a 5-min equilibration step for each.

##### 2.2.1.3 NP morphology characterization

Alg/Chit-NPs morphology was assessed using scanning electron microscopy (SEM). Briefly, 20 µL of Alg/Chit-NPs solution (135 μg/mL) with or without CXCL12-AF647 was placed onto a sample holder and left to dry in the biological safety cabinet for 2 h. The Alg/Chit-NPs were further metallized with a coating of palladium using an ion sputter coater (Anatech Hummer VI, NV, USA), and high-resolution images were taken at 30 kV using an S4700-Hitachi field emission SEM (Hitachi High-Technologies Canada, Toronto, ON, Canada).

#### 2.2.2 Release kinetics of CXCL12 under static conditions

##### 2.2.2.1 Alginate 1% and alginate:Matrigel hydrogel preparation

###### 2.2.2.1.1 Alginate 1% hydrogel preparation

To prepare an alginate solution, medical grade alginate powder was mixed with sterilized Milli-Q water at a final concentration of 1.5% (w/v). Effective mixing was guaranteed by exposing the solution for 12 h to vigorous magnetic stirring at room temperature. The solution was filtered using 45 µm filters and stored at 4°C. In a 96-multi-well low-binding plate (Corning^®^), alginate solution was mixed with water and CXCL12-AF647 solution with or without Alg/Chit-NPs to reach a final concentration of 1% alginate (w/v). A solution of CaCl_2_ (18 mM) with a final concentration of 1% (w/v) was used for gelation, and the mix was left 30 min at room temperature.

###### 2.2.2.1.2 Alginate:Matrigel (Alg:M) hydrogel preparation

A hydrogel of Alg:M (50:50) (w/w) was used to mimic the peri-tumoral space (Cavo et al., 2018). Matrigel^TM^ was thawed overnight at 4°C. Prior to forming the gels, 96-multi-well ultra-low binding plate (Greiner Bio-One) or culture chamber (for dynamic condition culture) were moved into ice to adapt to the Matrigel^TM^ temperature. Matrigel^TM^ was mixed with the alginate (1.5% w/v) for a final ratio of 50:50 (w/w), and then sterilized Milli-Q water was added with CXCL12-AF647 solution. The well plate or culture chamber were removed from the ice bath, a solution of CaCl_2_ (18 mM) was added to the mix, and the hydrogels were left for 30 min at room temperature for gelation.

##### 2.2.2.2 Release kinetics under static condition

For the release kinetic experiments, 4.3 µg of CXCL12-AF647 was added to the alginate before the calcium chloride and left for 5 min in the dark to agitate. The release kinetics of CXCL12-AF647 under static conditions was determined by fluorescence quantification using a fluorescence microplate reader (Safire2, Tecan US Inc., Morrisville, NC, United States) at an excitation wavelength of 650 nm, an emission wavelength of 665 nm, and a bandwidth of 5 nm.

**FIGURE 1:**
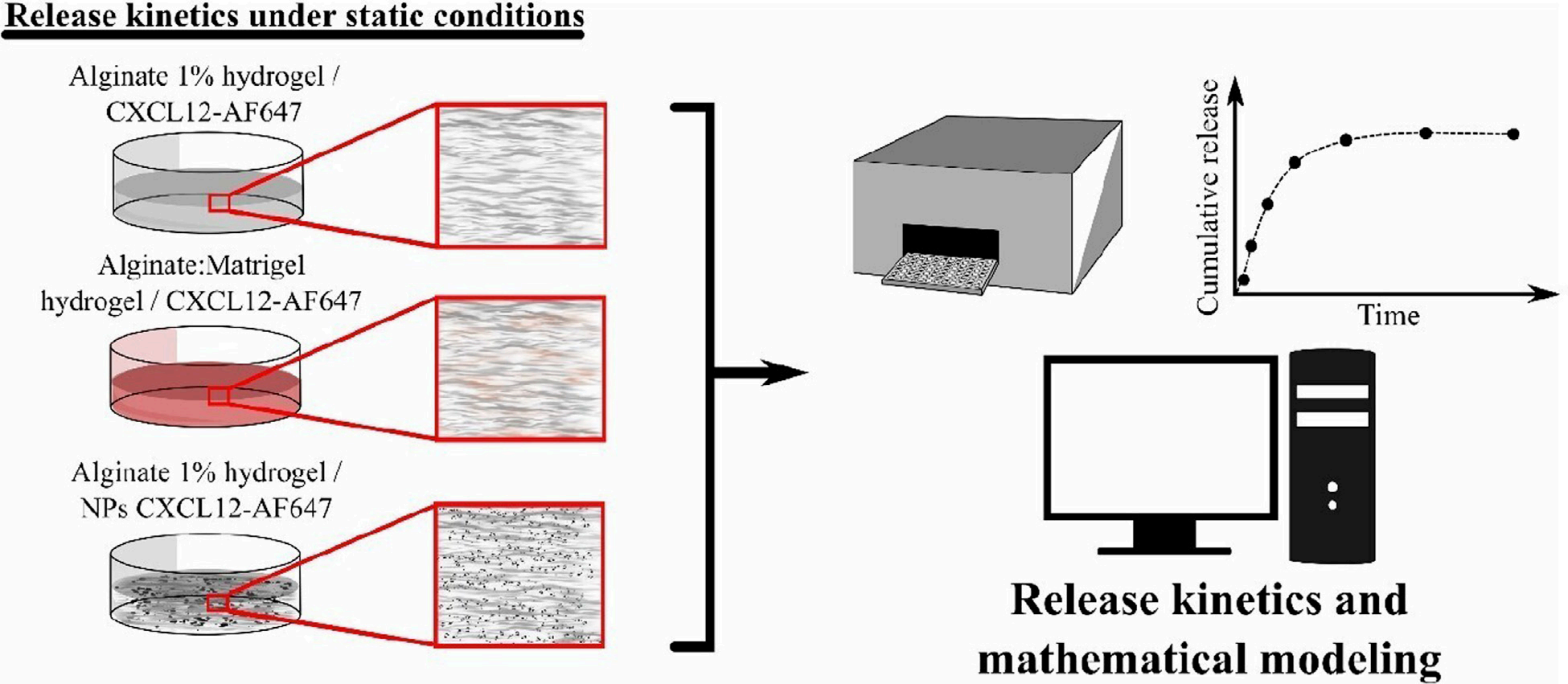
Schematic representation showing the kinetic release experiments under static conditions.

Bajetto et al. (2006) found that CXCL12 at a concentration of 100 ng/mL promotes the proliferation of primary GBM cells. We have previously shown that a percentage of the initial mass loaded in Alg/Chit-NPs remain encapsulated in the NPs (Gascon et al., 2020); therefore, to have 100 ng/mL of CXCL12 released, three concentrations (400 ng/mL, 800 ng/mL, and 1,600 ng/mL) of free CXCL12-AF647 or Alg/Chit-NPs loaded with CXCL12-AF647 were tested. Those concentrations represent the concentration range expected to be used for therapeutic application targeting glioblastoma cells (Gascon et al., 2020). CXCL12-AF647 or Alg/Chit-NPs loaded with CXCL12-AF647 were embedded and uniformly distributed in the alginate 1% (w/v) (Figure 1), whereas only free CXCL12-AF647 was used in Alg:M hydrogel, since we aimed to evaluate the molecule diffusivity in a hydrogel that imitates the tumor microenvironment. Both hydrogels were formed in ultra-low binding 96-multi-well plates for the kinetic release study. To the well plates was added 100 μL tempered DMEM (without phenol red) at 37°C , which was then incubated in a humidified cell-culture incubator (Forma Series II, water jacket CO_2_ incubator, Thermo Electron Corporation, Gormley, ON, United States) at 37°C for different time points between 0 and 168 h under static release conditions. After each time point (0, 0.5, 1, 2, 4, 6, 8, 24, 48, 72, 120, 144, and 168 h), the complete supernatants were collected and transferred to ultra-low binding microcentrifuge tubes to avoid non-specific adsorption for fluorescence quantification, and a fresh DMEM solution was added. The amount of released CXCL12-AF647 was determined as the cumulative mass release described as follows:

$$\frac{M_{t}}{M_{\infty }}=100\left(\frac{\sum_{i=0}^{t}M_{i}}{M_{initial}}\right),$$

where $M_{t}$ is the total mass of the CXCL12-AF647 released at t time, $M_{\infty }$ is the initial mass loaded, and $M_{i}$ is the cumulative mass of the molecule released at t time.

##### 2.2.2.3 Mathematical modeling and parameter estimation for static condition of CXCL12-AF647 release

###### 2.2.2.3.1 Korsmeyer–Peppas representation

To estimate the driving transport phenomenon occurring in respect of the delivery system geometry, we modeled the percentage of the cumulative mass released for the first 6 h using the Korsmeyer–Peppas representation (Korsmeyer et al., 1983):

$$\frac{M_{t}}{M_{\infty }}=at^{n}.$$

The values of the exponential term “n” corresponding to the slope of the linearized equation—of interest for deciphering the main driving release mechanism—were determined using a standard linear regression over the cumulative release percentage.

###### 2.2.2.3.2 Fickian-based mechanistic modeling

The mathematical framework was developed considering the following assumptions:

• Hydrogels are cylindrical.
• CXCL12-AF647 (free or loaded into Alg/Chit-NPs) is uniformly distributed in the hydrogels with a concentration below saturation (monolithic dispersion).
• Diffusion is the major mass transport phenomenon occurring and was considered isotropic in the axial dimension and null in the radial dimension.
• The effective diffusion coefficient is constant over time and space.
• The electrostatic interactions between CXCL12-AF647 (pI of ∼10) (positively charged at physiological pH) and alginate (negatively charged) are responsible for releasing CXCL12 at the hydrogel interface.

From these assumptions, the mathematical framework was developed based on Fick’s second law of diffusion for cylindrical coordinates assuming constant diffusion:

$$\frac{\partial C_{CXCL12}}{\partial t}=D_{eff}\left(\frac{\partial^{2}C_{CXCL12}}{\partial z^{2}}\right).$$

With the following initial conditions,

$$At\;t=0,\forall z\in \left[0,L\right]\to {\left(C_{CXCL12}\right)}_{t=0}=C_{initial}.$$

Furthermore, Newman and Robin’s boundary conditions used as the mass flux at the basis was null, and the release at the superior surface of the hydrogel was driven by the concentration gradient between the molecule concentration and the pseudo-equilibrium concentration influenced by the electrostatic interactions between the solute and the hydrogels:

$${\left(\frac{\partial C_{CXCL12}}{\partial z}\right)}_{z=0}=0$$

and

$${\left(\frac{\partial C_{CXCL12}}{\partial z}\right)}_{z=L}=k\;\left(C_{cxcl12\;|L}-C_{eq}\right).$$

A finite difference approach was used to solve the model in respect to time and space. Briefly, an implicit Crank–Nicolson scheme was used due to its numerical stability. The total amount of CXCL12-AF647 released at “t” time was estimated using a trapezoidal method:

$$M_{t}=M_{\infty }-\left[{\pi R}^{2}\sum_{j=0}^{J}\frac{\left(C_{j+1}^{t}+C_{j}^{t}\right)z}{2}\right],$$

where z is the position step defined as the overall size divided by the number of nods where the function is evaluated. In this case, cylindrical gels with a diameter of 6.5 mm and a height of 5 mm were discretized into 30 nods (space steps of 0.167 µm). The numerical integration time was set at 300 s. Once the mathematical framework was set, a genetic algorithm was used to estimate the diffusion coefficient (D_eff_) and the overall mass transfer coefficient at the surface (k) as previously described (Lauzon et al., 2018) from the release kinetics data.

#### 2.2.3 Release kinetics under perfusion

##### 2.2.3.1 Bioreactor design

**FIGURE 2:**
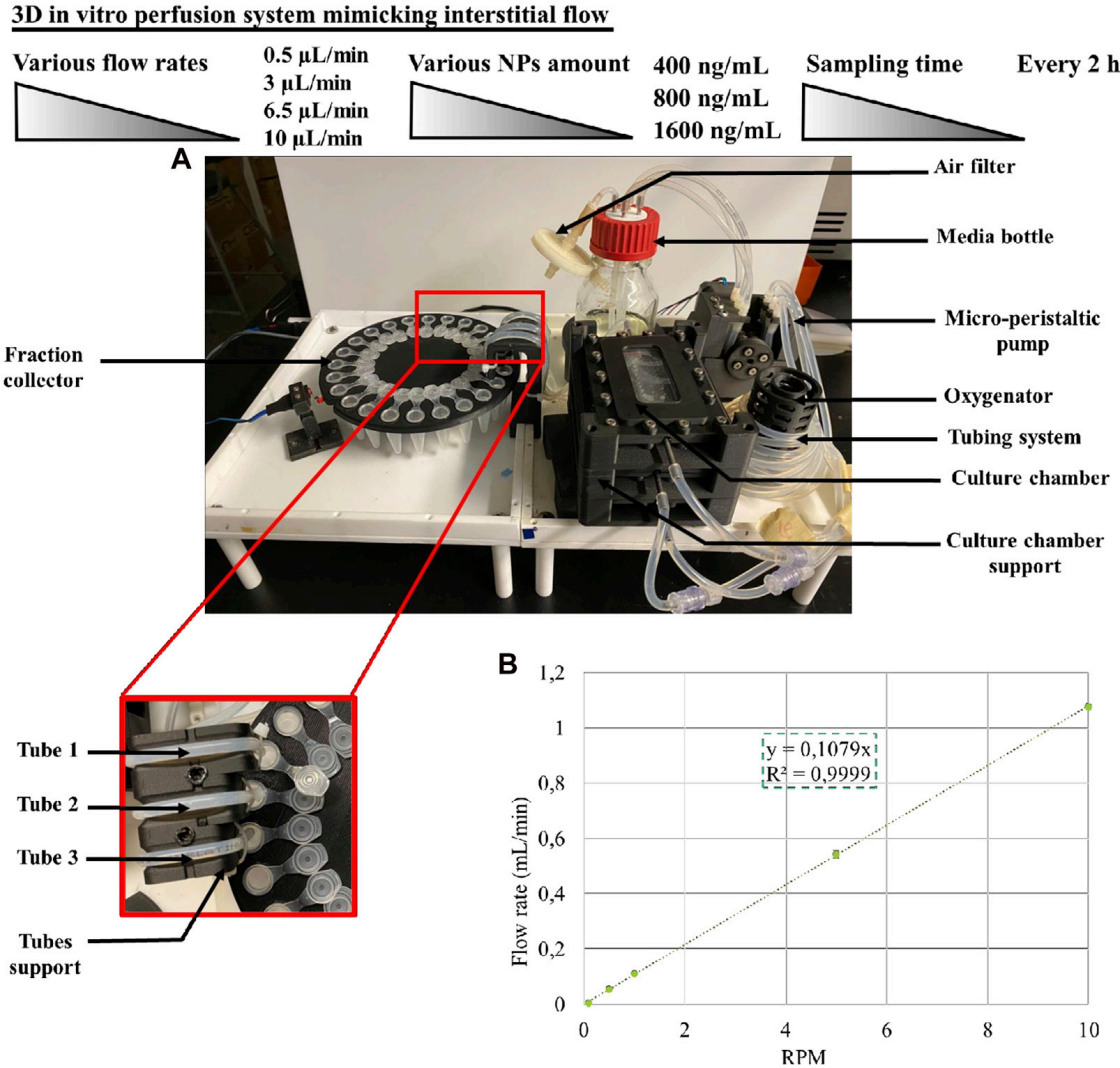
**(A)** Perfusion bioreactor and the fraction collector assembly. **(B)** Calibration curve of the micro-peristaltic pump.

We developed a perfusion bioreactor to study the release kinetic of CXCL12-AF647 under simulated physiological stimulation aiming to mimic the brain IFF (Figure 2A). The perfusion bioreactor system was designed to concomitantly provide dynamic continuous perfusion to a 3D scaffold with the aim of mimicking the physiological loads. This will provide a better understanding of the effect of the interstitial fluid on the molecule release under *in vitro* conditions that recapitulate *in vivo* conditions (T°, ${CO}_{2},O_{2}$ , and IFF).

From the media bottle to the oxygenator, 250 mL of fresh DMEM supplemented with 1% (v/v) antibiotics was pumped through L/S 14 platinum-cured silicone tubing (Masterflex^®^) by a custom-designed micro-peristaltic pump driven by a precise stepper motor. Since the peristaltic pump is a positive-displacement system, the flow rate is proportional to the rotational speed of the pump rotor.

We performed a linear calibration to determine the rotational speed of the pump rotor to achieve the desired flow rates to be used for the kinetic release of CXCL12-AF647 in this study. From 0°C to 80°C, density was determined using the Thermo Library (Thermodynamics and Phase Equilibrium component of Chemical Engineering Design Library) (Bell, 2021) at atmospheric pressure (the solution used for the perfusion bioreactor calibration). A polynomial curve was obtained from the density of water and the temperature. Subsequently, at different RPM (0.1, 0.5, 1, 5, and 10) for various periods from 10 min to 2 h, the mass of water was collected from the three tubes (Figure 2A). The results are representative of three independent experiments for each tube. The flow rate was determined from the weight of water collected divided by the density and by the sampling time as follows:

$$flow\;\left(mL/\min\right)=\frac{mass\;of\;water\;\left(g\right)}{density\;\left(\frac{g}{mL}\right)∙time\;\left(\min\right)}.$$

**FIGURE 3:**
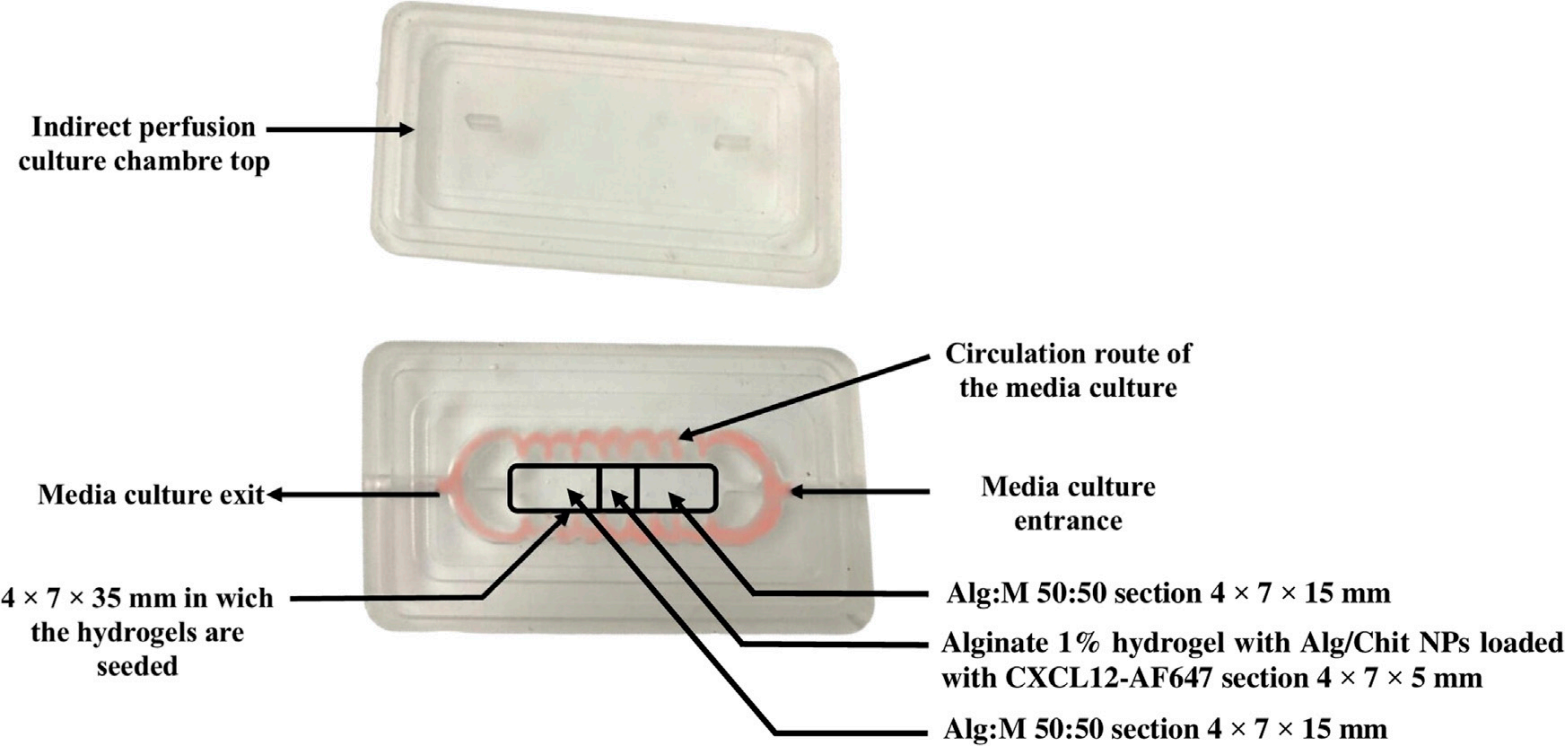
Image of the PDMS culture chamber and its lid. The circulation route of the fluid is shown in red. The framed sections depict the position and composition of the gels.

Finally, the average of flow rates was calculated, and the calibration curve was obtained using a linear regression (Figure 2B). For the duration of the experiment (maximum 72–120 h depending on the rate) at different rates (0.5 μL/min, 3 μL/min, 6.5 μL/min, and 10 μL/min), the media was perfused through the culture chambers (Figure 3). All pieces of the bioreactor (the lid of the culture medium bottle, the oxygenator, the micro-peristaltic pump, the fraction collector, and the support for the tubing) were designed and 3D-printed in carbon fiber-reinforced nylon. The perfusion bioreactor is actuated through a microcontroller board and is connected to a computer for control (pump rotation, stirring speed, duration of the fraction collection, and angle of rotation), monitoring, and data logging.

##### 2.2.3.2 Culture chamber design and fabrication

The culture chambers were designed to perform two types of perfusion: direct and indirect. We focused our work on indirect perfusion to allow the CXCL12-AF647 to seep into the Alg:M in dynamic conditions. The design of the culture chamber was determined by our team. Briefly, the culture chambers were prepared using polydimethylsiloxane (PDMS), since it provides a suitable environment for cell culture and has the advantage of being optically clear to allow in-line microscopic monitoring. In a disposable plastic container, the polymer solution was mixed vigorously with the crosslinking agent for 2 min for a final ratio of 10 (polymer):1 (crosslinker) (v/v) following the manufacturer’s instructions. A vacuum cycle was performed for 1 h to degas the polymer at −22 kPa (pressure-gauge). Using dry molds sprayed with silicon grease, the PDMS-crosslinker solution was poured, and the molds were transferred to the oven at 80°C for 4 h. They were left to cool overnight, and the PDMS culture chambers were gently unmolded (Figure 3).

Before use, the culture chambers and their lids were sterilized by immersion in 70% (v/v) ethanol for 5 h, rinsed with sterile distilled water, and left to dry in the biological safety cabinet.

##### 2.2.3.3 Release kinetics

**FIGURE 4:**
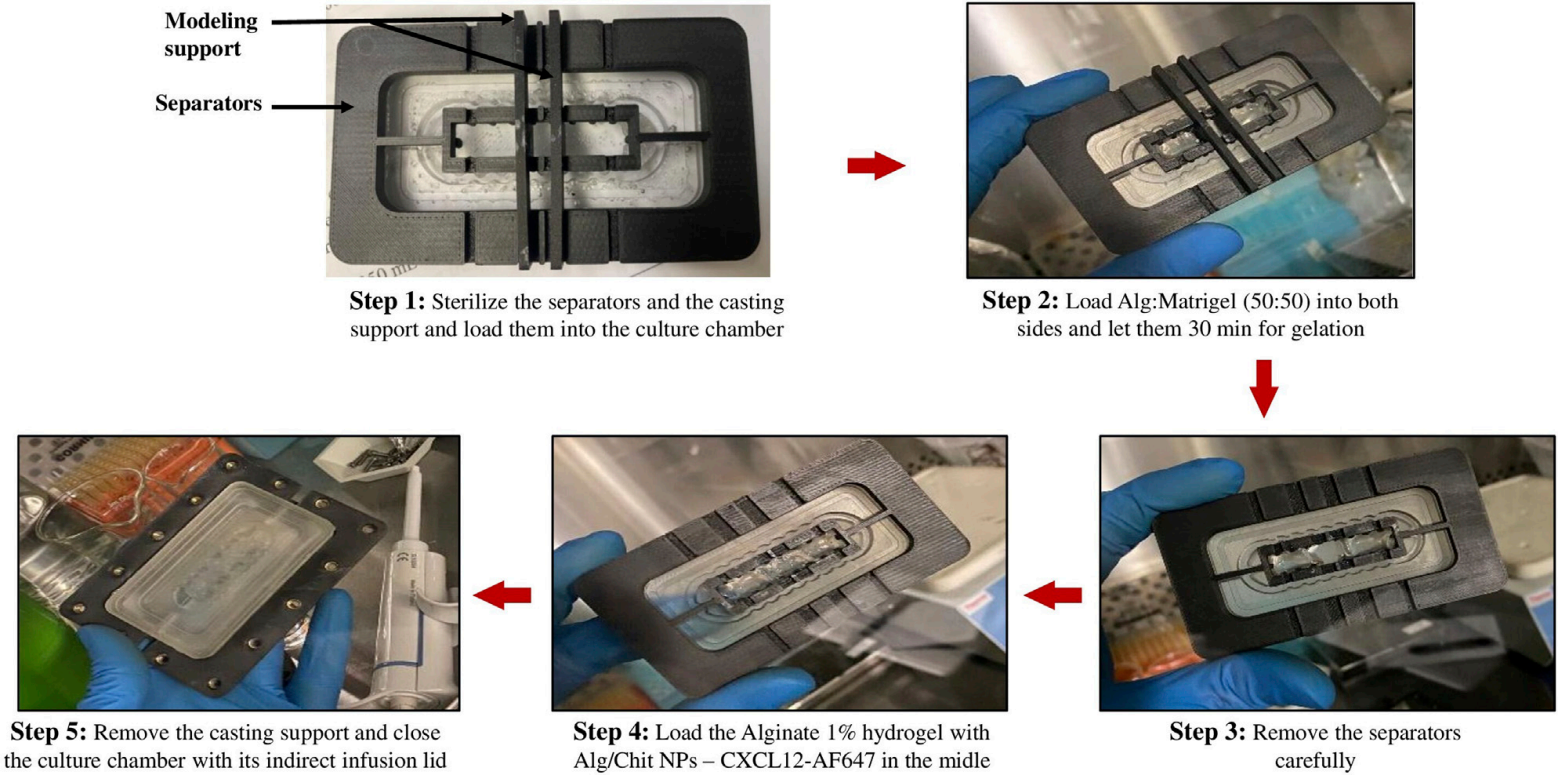
Images of the different steps for gel preparation using the molding support and separators.

Using molding support separators (Figure 4), Alg:M 50:50 (v/v) hydrogels were first jellified in the culture chamber, then the separators positioned on a jig were removed, and alginate 1% hydrogel containing Alg/Chit-NPs loaded with CXCL12-AF647 was jellified in the middle. This approach allows precise control of the dimensions of each gel section and ensures their direct and continuous contact.

The gels were formed by the successive deposition of four identical layers (same gel volume) to ensure good gelation. Each layer was left for 10 min for gelation and 40 min at the end. The chambers were closed and held in the bioreactor using culture chamber supports, thus ensuring complete sealing (Figure 4). The perfusion bioreactor was coupled to a fraction collector. The fractions of DMEM with the CXCL12-AF647 released in the surrounding moving fluid were collected at the exit of the culture chamber every 2 h for fluorescence quantification, for a total duration of 120 h. CXCL12-AF647 concentrations of 400 ng/mL, 800 ng/mL, and 1,600 ng/mL were assessed at four flow rates that recapitulate the different IFF rates found in the brain. The amount of CXCL12-AF647 released was determined as previously described by fluorescence quantification.

##### 2.2.3.4 Fluorescence scans and CXCL12 distribution in the gels

Concentrated CXCL12-AF647 was detected when encapsulated in NPs, allowing scans of the overall culture chamber section at t = 0, t = 24 h, and the end of each experiment (t = 120 h for 0.5 μL/min and 3 μL/min, t = 72 h for 6.5 μL/min and t = 48) using EVOS™ FL Auto Imaging System with a CY5 light cube (Life Technologies), and a custom-made adaptor for the culture chamber. The scans were assessed without interrupting the release kinetics.

At the end of the experiment, the different sections of the gels (Alg:M in the flow direction, Alg:M in the opposite flow direction, and alginate 1% containing Alg/Chit-NPs loaded with the molecule) were recovered individually to quantify the remaining CXCL12-AF647 in each gel. A dissociation solution (pH = 7.4) containing 16.1 mg/mL of sodium citrate, 8.7 mg/mL of NaCl, and 10.2 mg/mL of ethylenediaminetetraacetic acid (EDTA) was prepared. Each part of the gels was placed in 1.5-mL low-bind microtubes, and 350 µL of the solution was added. The tubes were left for 30 min for incubation under medium stirring and were then centrifuged at 20,000 × g for 30 min at 10°C. The CXCL12-AF647 in the supernatants was then quantified. Consequently, 350 µL of Tris-EDTA buffer was added to the solid recovered from the alginate 1% containing the Alg/Chit-NPs after centrifugation for NP dissociation and left for 30 min for incubation. The solution was centrifuged at 20,000 × g for 30 min at 10°C, and the CXCL12-AF647 in the supernatant was quantified as described previously.

#### 2.2.4 Statistical analysis

Statistical analyses were assessed on Microsoft Excel (Analysis tool pack) using analysis of variance (one-way ANOVA) for at least three independent experiments performed in triplicate. Only differences of *p* < 0.05 were considered statistically significant.

## 3 Results

### 3.1 Nanoparticle synthesis and stability

**FIGURE 5:**
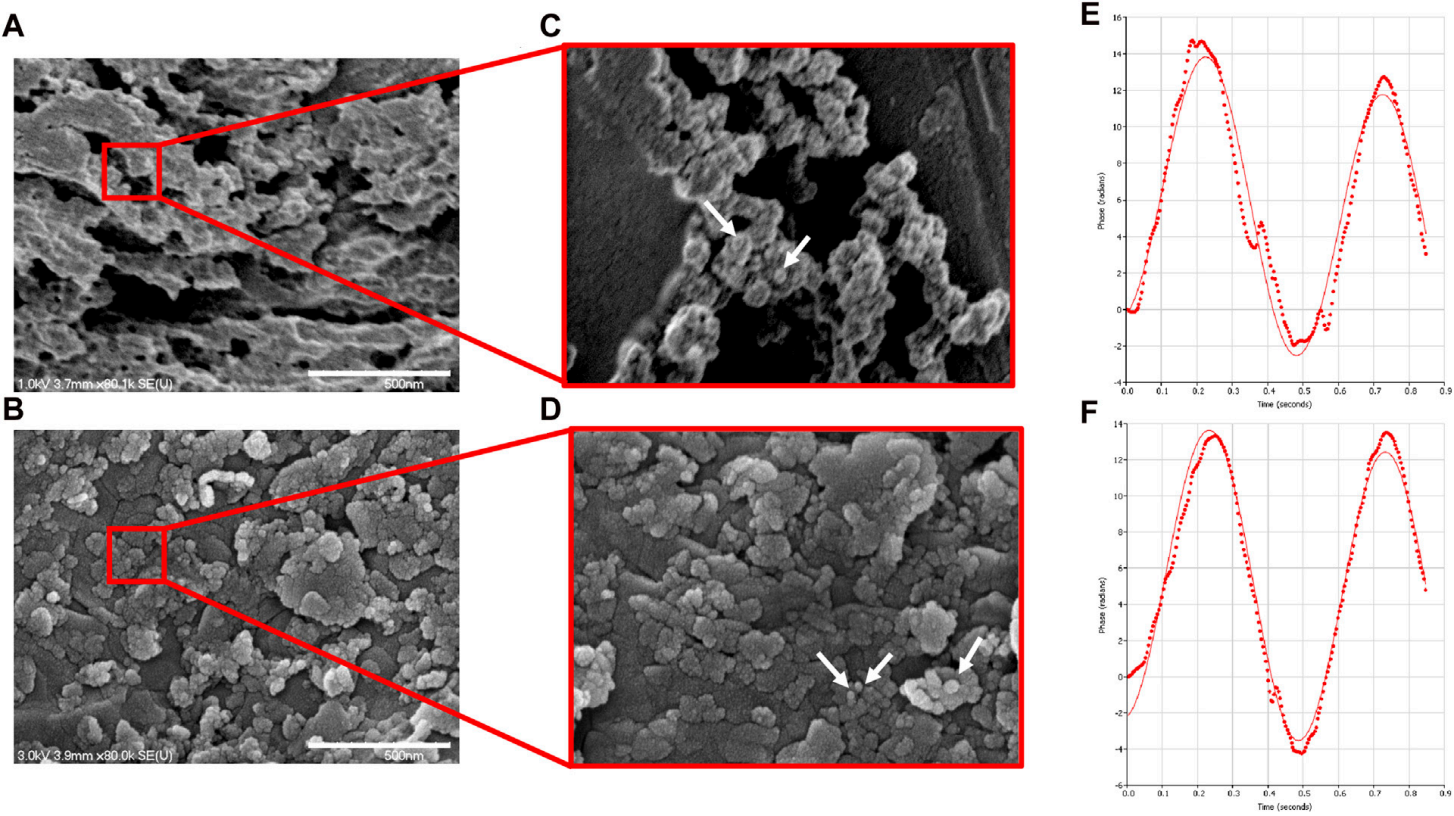
Representative SEM image of **(A)** empty NPs and **(B)** NPs containing CXCL12-AF647 (0.744 μg/mg NPs). **(C,D)** Zoom regions highlighted by red squares for NPs and NPs-CXCL12-AF647, respectively. **(E,F)** Representative graphs of the zeta potential measurement for empty Alg/Chit-NPs and NPs-CXCL12-AF647, respectively.

NP morphology with and without CXCL12-AF647 was first characterized by SEM observation (Figure 5). SEM analyses of dehydrated Alg/Chit-NPs indicated the presence of large aggregates (Figures 5A, B). The presence of CXCL12-AF647 did not affect the Alg/Chit-NPs’ spherical morphology (Figures 5C, D) as previously reported (Gascon et al., 2020). The zeta potential, which is the surface charge responsible for a particle’s stability through the electronic repulsion between them, was also measured for Alg/Chit-NPs loaded or not loaded with CXCL12. The results of the Alg/Chit-NPs with and without CXCL12-AF647 were 41.73 mV and 40.03 mV, respectively (Figures 5E, F), suggesting that the encapsulation of CXCL12 did not affect NP stability.

### 3.2 Release kinetics under static condition, mathematical modeling, and parameter estimation

#### 3.2.1 Release kinetics of CXCL12-AF647 from alginate 1% and Alg:M (50:50) hydrogels and their mathematical modeling

**FIGURE 6:**
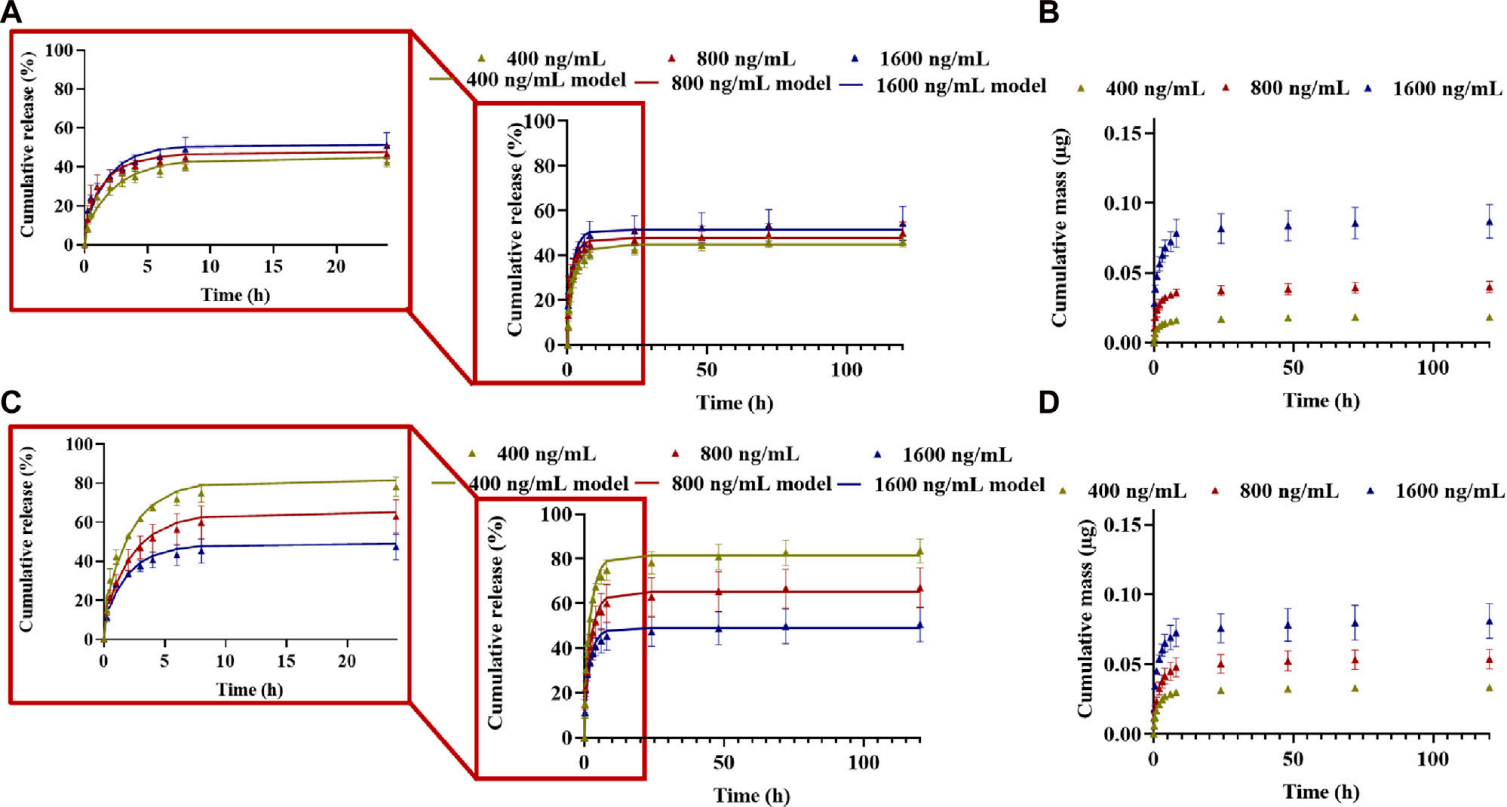
Cumulative release kinetics and mathematical modeling results for different initial mass loaded (400 ng/mL, 800 ng/mL, and 1,600 ng/mL) in **(ALB)** alginate 1% hydrogel and **(C,D)** A:M (50:50) hydrogel. **(A,C)** Cumulative percentage release; **(B,D)** cumulative absolute for different mass loadings. Results are representative of at least three independent experiments performed in triplicate.

The release of CXCL12-AF647 (pI of ∼10) from alginate 1% hydrogel was quantified within 120 h (5 days) using a standard curve obtained by fluorescence measurement (data not shown). For all initial mass loadings of CXCL12 (0.04, 0.08, and 0.16 µg), a burst release was generally observed in the first 3 h due to the fast diffusion of the molecule located at the surface of the hydrogels. The burst release was followed by a sustained release for up to 120 h, typical of a diffusion-based system, and a pseudo-plateau was reached after 72 h (Figures 6A, B). As the initial mass load increased, the total cumulative release percentage increased, and total mass releases were 46.07% ± 2.26 (0.018 µg ± 0.001) for 400 ng/mL, up to 50.04% ± 4.94 (0.040 µg ± 0.003) for 800 ng/mL, and 54.31% ± 7.51 (0.086 µg ± 0.012) for 1,600 ng/mL. For the three mass loadings, the pseudo-plateau was reached without a complete release of the CXCL12 molecules.

For its release from Alg:M (50:50) hydrogel, the cumulative percentage and total mass release of CXCL12-AF647 were 83.52% ± 5.43 (0.033 µg ± 0.002) for 400 ng/mL, 67.07% ± 8.83 (0.053 µg ± 0.007) for 800 ng/mL, and 50.59% ± 7.72 (0.08 µg ± 0.01) for 1,600 ng/mL after 120 h (Figures 6C, D). The percentage of the cumulative mass released increased as the initial mass of CXCL12-AF647 loaded increased. This is consistent with a diffusion-based system in which a gradient of CXCL12 is created when the initial loading mass is increased. For all the experiment conditions, the pseudo-plateau was reached without a complete release of the molecule, suggesting the presence of strong electrostatic interactions.

We then first modeled the release kinetics data using the Korsmeyer–Peppas representation (Korsmeyer et al., 1983) to estimate the driving mass transport phenomena. The values of the exponential term “n,” corresponding to the slope of the linearized equation, were determined using a standard linear regression over the cumulative release percentage data plot on a logarithmic scale (Tables 1, 2). For a system with cylindrical geometry, a value of *n* = 0.45 shows that CXCL12-AF647 release is controlled by Fickian diffusion, whereas *n* = 0.89 suggests that CXCL12 release mainly occurs by an erosion mechanism. A value of 0.45 < n < 0.89 describes the release as anomalous, which means that both diffusion and erosion are taking place (Korsmeyer et al., 1983). The results showed that for 400 ng/mL, *n* = 0.45, indicating Fickian diffusion as the predominant mass transport phenomenon. For 800 ng/mL and 1,600 ng/mL, the values of “n” were below 0.45, also indicating a Fickian diffusion. Similar results were obtained with CXCL12 release from NPs due to the presence of electrostatic interactions between the negative charge of the alginate and the positive charge of CXCL12 (Gascon et al., 2020).

**TABLE 1:** Mechanistic parameter estimation of the mathematical framework for CXCL12-AF647 released from alginate 1% hydrogel (±STD).

Initial mass loading µg of CXCL12-AF647/mL	Korsmeyer–Peppas model		Fick model		
	Exponential parameter	R^2^	D_eff_ $m^{2}.s^{-1}\times 10^{-9}$	K $m.s^{-1}\times 10^{-5}$	R^2^
0.4	0.45 ± 0.07	0.92 ± 0.08	1.04 ± 0.03	4.13 ± 0.05	0.97 ± 0.01
0.8	0.37 ± 0.17	0.90 ± 0.05	1.98 ± 0.03	4.59 ± 0.04	0.96 ± 0.02
1.6	0.29 ± 0.02	0.979 ± 0.004	1.40 ± 0.04	1.190 ± 0.003	0.965 ± 0.004

**TABLE 2:** Mechanistic parameter estimation of the mathematical framework for CXCL12-AF647 release from Alg:M (50:50) (±STD).

Initial mass loading µg of CXCL12-AF647/mL	Korsmeyer–Peppas model		Fick model		
	Exponential parameter	R^2^	D_eff_ $m^{2}.s^{-1}\times 10^{-9}$	*K* $m.s^{-1}\times 10^{-5}$	R^2^
0.4	0.48 ± 0.15	0.93 ± 0.02	1.2 ± 0.3	6.75 ± 0.04	0.978 ± 0.005
0.8	0.43 ± 0.12	0.977 ± 0.006	1.1 ± 0.2	4.58 ± 0.04	0.98 ± 0.02
1.6	0.39 ± 0.05	0.91 ± 0.04	1.4 ± 0.4	2.40 ± 0.01	0.97 ± 0.01

Since the predominant releasing mechanism of CXCL12 was diffusion, we used Fick’s second law with Newman and Robin boundary conditions that consider the interactions between the molecule of interest and the delivery system to model the release kinetics data (% cumulative mass release). An evolutionary algorithm was used to estimate the model parameters: the effective diffusion (D_eff_) and the overall mass transfer coefficient (k) at the surface. For alginate and Alg:M hydrogels, the R^2^ ∼0.97 results proved that the model fits well with the experimental data for both hydrogels. The values of D_eff_ were in the same order of magnitude (∼1 × 10^−9^ $m^{2}.s^{-1}$ ) (Tables 1, 2), suggesting that the diffusion of CXCL12-AF647 is independent of the initial mass loading, as previously observed with the NPs (Gascon et al., 2020), thus confirming the choice to use a constant diffusion coefficient in the mathematical framework. Furthermore, for alginate 1% hydrogel, no difference was observed of the overall mass transport coefficient at the surface as the initial mass of CXCL12-AF647 increased, whereas for Alg:M (50:50) hydrogel, we found that k decreases as the initial mass loaded increases, suggesting that there is resistance to the CXCL12 release at the surface of the hydrogels.

#### 3.2.2 Release kinetics of CXCL12-AF647 encapsulated in Alg/Chit-NPs and loaded into alginate 1% hydrogel and their mathematical modeling

For better control of the CXCL12 burst release observed in the first hours, we studied the release kinetics of Alg/Chit-NPs loaded with CXCL12-AF647 from alginate 1% hydrogel. As we previously reported, encapsulation of CXCL12 into Alg/Chit-NPs was nearly independent of the chemokine initial mass loadings tested. Therefore, we decided to work with 1.490 μg/mg NP formulation as this provides high encapsulation efficiency and an overall release of ∼60% of the initial mass loaded (Gascon et al., 2020). To obtain CXCL12 concentrations of 400 ng/mL, 800 ng/mL, and 1,600 ng/mL in the hydrogels, different volumes from Alg/Chit-NPs-CXCL12 solution corresponding to CXCL12 mass of 0.07 µg, 0.13 µg, and 0.26 µg, respectively, were incorporated into the hydrogels. In addition, as the NPs retain 40% of the CXCL12, an excess of CXCL12 of 40% was administrated. For all the experimental conditions, the release of the molecule was quantified within 168 h (7 days).

**FIGURE 7:**
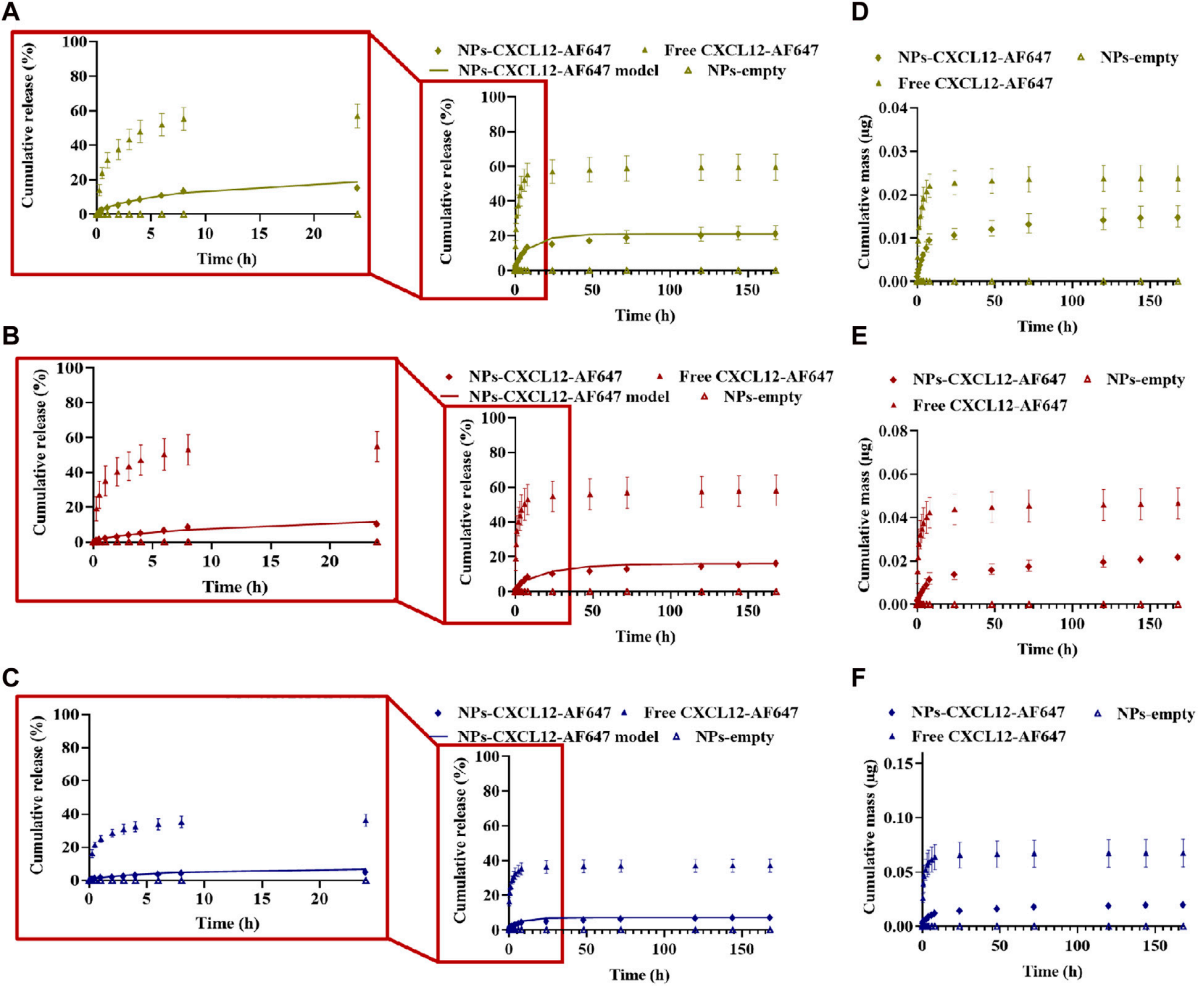
**(A–C)** Cumulative release kinetics and mathematical modeling results of different initial mass of CXCL12-AF647 loaded in Alg/Chit-NPs from alginate 1% hydrogel with zoom regions highlighted by red squares for the first 24-h release kinetics. **(D–F)** Cumulative absolute mass released. Results are representative of at least three independent experiments performed in triplicate.

Unlike the results observed with free CXCL12-AF647, the release of the chemokine when encapsulated in NPs was more sustained, and the burst released was much less pronounced in the first hours of the study for all the tested experimental conditions (Figures 7A–C). The release of CXCL12-AF647 encapsulated in NPs was also slower than when its free form was loaded into the hydrogel. Indeed, 21.77% ± 4.09 (0.015 µg ± 0.002), 16.85% ± 1.46 (0.022 µg ± 0.002), and 7.75% ± 1.36 (0.021 µg ± 0.003) of the CXCL12 encapsulated in the NPs were released after 168 h compared to 42.46% ± 7.54 (0.016 µg ± 0.003), 58.14% ± 8.92 (0.046 µg ± 0.007), and 37.14% ± 9.96 (0.06 µg ± 0.01) of free CXCL12-AF647 for 400 ng/mL, 800 ng/mL, and 1,600 ng/mL, respectively (Figure 7). Furthermore, the CXCL12-AF647 needs more time to first diffuse from the NPs to the hydrogel, and then from the hydrogel to the culture media. When free CXCL12-AF647 was released from alginate 1% hydrogel, the molecule interacted with the alginate chains and was released directly into the culture media; the release was driven by the concentration gradient between CXCL12 and the pseudo-equilibrium concentration, as previously described (Gascon et al., 2020). In contrast, the loaded CXCL12-AF647 in Alg/Chit-NPs embedded into the alginate 1% hydrogel must be diffused from the NPs to the hydrogel before being released into the culture media. This implicates the presence of more interactions at the release surface between the NPs and the alginate hydrogel.

Moreover, the results of the release kinetics of NPs-CXCL12-AF647 from 1% alginate were modeled to evaluate the mass transport phenomenon that occurred, as described earlier. Korsmeyer–Peppas representation was used, and the values of n for the concentrations 400 ng/mL and 800 ng/mL were between 0.45 and 0.89, suggesting that the transport phenomenon of CXCL12 is primarily coupled to diffusion. For 1,600 ng/mL, the main mechanism driving CXCL12 release is diffusion with the presence of interactions, since n was lower than 0.45. The values of the effective diffusion and the overall mass transfer coefficient respecting the initial mass loaded were determined using Fick’s second law. The results showed that the model was well fitted to the experimental data with a high coefficient of determination. The mathematical framework was solved as described earlier to estimate the model parameters (Table 3). The values of D_eff_ and k were in the same order of magnitude between the same experimental conditions. However, the D_eff_ was a lower order of magnitude compared with the values obtained for free CXCL12. Those results are in accordance with the experimental results in which the molecule takes more time to diffuse within the NPs toward the hydrogel, possibly due to the presence of strong electrostatic interactions between the molecule and the negative charge of the alginate composing the NPs and hydrogel, in addition to more resistance at the interface between the NPs and the surrounding hydrogel.

**TABLE 3:** Mechanistic parameter estimation of the mathematical framework (±STD) for the release of CXCL12-AF647 encapsulated in Alg/Chit-NPs and loaded into alginate 1% hydrogel.

Initial mass loading µg of CXCL12-AF647/mL	Korsmeyer–Peppas model		Fick model		
	Exponential parameter	R^2^	D_eff_ $m^{2}.s^{-1}\times 10^{-10}$	K $m\;.s^{-1}\times 10^{-6}$	R^2^
0.4	0.54 ± 0.11	0.987 ± 0.004	2.4 ± 0.6	2.98 ± 1.08	0.97 ± 0.01
0.8	0.51 ± 0.17	0.97 ± 0.02	1.28 ± 0.62	1.28 ± 0.38	0.954 ± 0.008
1.6	0.31 ± 0.04	0.981 ± 0.005	3.91 ± 2.16	3.05 ± 1.23	0.94 ± 0.03

### 3.3 Release kinetics of Alg/Chit-NPs-CXCL12-AF647 under perfusion

#### 3.3.1 Release kinetics under dynamic conditions and CXCL12-AF647 distribution in the hydrogels

To study CXCL12-AF647 kinetics release under perfusion conditions that mimic the brain IFF, three initial mass loadings (0.08 µg, 0.16 µg, and 0.32 µg) were investigated under four different flow rates. The release of Alg/Chit-NPs-CXCL12-AF647 from 1% alginate hydrogel was quantified over various time periods until it was no longer found in the collected media.

**FIGURE 8:**
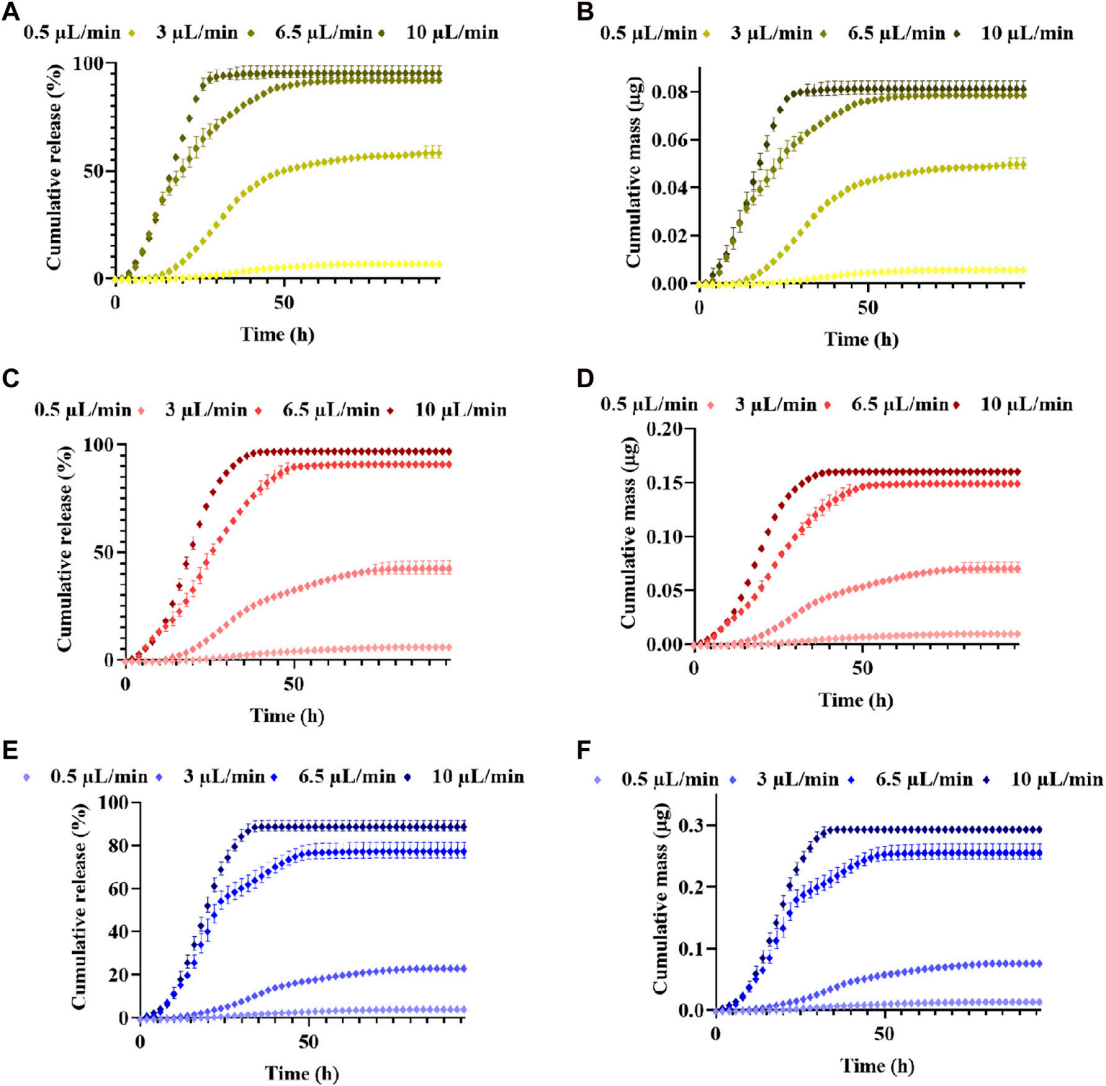
Release kinetics and cumulative mass released ±SD of CXCL12-AF647 from 1% alginate hydrogels for different mass loadings of CXCL12-AF647 **(ALB)** 400 ng/mL, **(C,D)** 800 ng/mL, and **(E,F)** 1,600 ng/mL under different flow rates of 0.5 μL/min, 3 μL/min, 6.5 μL/min, and 10 μL/min. Results are representative of at least three independent experiments.

For the concentration 400 ng/mL, an initial mass loading of 0.0855 µg of CXCL12-AF647 was used. Under 0.5 μL/min, 3 μL/min, 6.5 μL/min, and 10 μL/min, 7.3% ± 0.7 (0.0062 µg ± 0.0006), 58.7% ± 2.7 (0.050 µg ± 0.002), 92.5% ± 1.3 (0.079 µg ± 0.001), and 95.9% ± 2.8 (0.081 µg ± 0.002) of CXCL12-AF647 were released, respectively (Figures 8A, B). From 0.5 μL/min, 3 μL/min, 6.5 μL/min to 10 μL/min, CXCL12-AF647 quantification started after 10 h, 6 h, 4 h, and 2 h, respectively, which can be explained by the dead volume of the media culture present in the pipe going from the culture chamber into the fraction collector. Pseudo-plateaus were reached more rapidly as the flow rate increased. They were attained after 86 h, 96 h, 72 h, and 48 h for 0.5 μL/min, 3 μL/min, 6.5 μL/min, and 10 μL/min, respectively, without a complete release of CXCL12-AF647. We obtained the same release profile with a concentration of 800 ng/mL in which an initial mass loading of 0.165 µg of CXCL12-AF647 was used. Cumulative percentages of 6.7% ± 0.4 (0.011 µg ± 0.001), 43.02% ± 3.06 (0.071 µg ± 0.005), 91.5% ± 2.1 (0.150 µg ± 0.001), and 97.5% ± 1.3 (0.161 µg ± 0.002) from the initial mass loading of 0.16 µg were released under 0.5 μL/min, 3 μL/min, 6.5 μL/min, and 10 μL/min flow rates, respectively (Figures 8C, D). Pseudo-plateaus were reached similarly to the first mass loading, and a high percentage of CXCL12-AF647 remained trapped in the hydrogels under 0.5 μL/min compared to 3 μL/min. Under those flow rates, the release percentages of the CXCL12-AF647 indicated that a convection phenomenon was occurring, whereas the remaining CXCL12-AF647 was still inside the hydrogels. When the initial mass loaded was increased to 0.331 µg, percentages of 4.5% ± 0.9 (0.015 µg ± 0.003), 23.5% ± 1.1 (0.077 µg ± 0.003), 77.8% ± 3.7 (0.25 µg ± 0.01), and 89.2% ± 2.5 (0.295 µg ± 0.008) were released under 0.5 μL/min, 3 μL/min, 6.5 μL/min, and 10 μL/min, respectively (Figures 8E, F) The results show that using dynamic culture at high flow rates (6.5 μL/min and 10 μL/min), a release of 75%–∼90% from the initial mass loadings was obtained, suggesting that convection is the major transport phenomenon.

Furthermore, at the end of the release kinetic study, we analyzed the CXCL12 content of each gel to validate whether the trapped CXCL12-AF647 remained in the 1% alginate hydrogel containing Alg/Chit-NPs or was diffused in the Alg:M (50:50) hydrogel that mimics the peritumoral space. We found that the cumulative mass release plus the remaining CXCL12-AF647 in the different parts of the hydrogels (Alg:M in the flow direction, alginate 1%, Alg/Chit-NPs, and Alg:M against flow direction) accounted for ∼100% of the total initial mass encapsulated.

**FIGURE 9:**
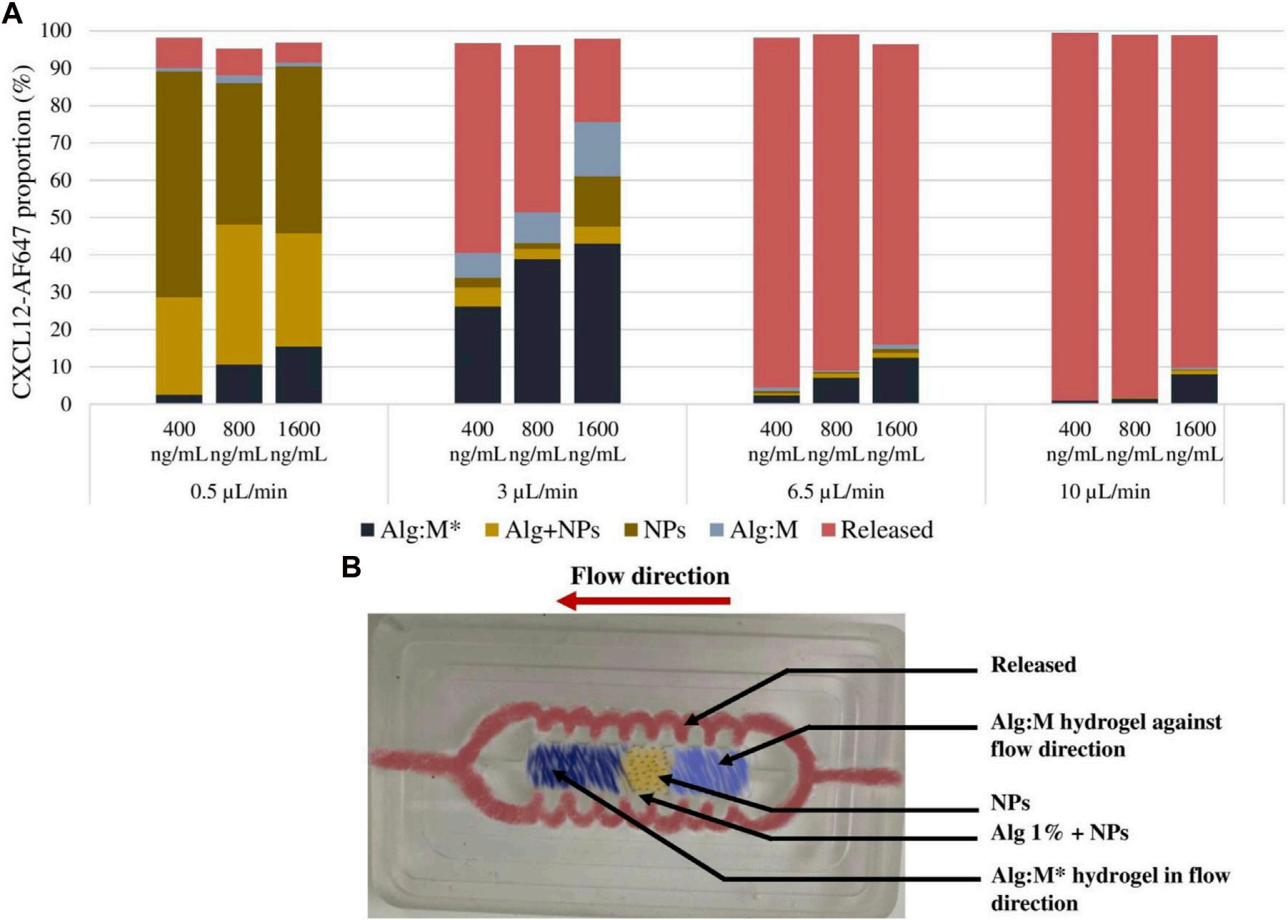
**(A)** Distribution analysis of the remaining CXCL12-AF647 in the different sections of the hydrogels for the different concentration (400 ng/mL, 800 ng/mL, and 1600 ng/mL) and under different flow rates (0.5 µL/min, 3 µL/min, 6.5 µL/min, and 10 µL/min). **(B)** Image of the culture chamber illustrating the different compartments of the gel and the flow circulation route.

At a low flow rate (0.5 μL/min), the molecule was mainly trapped in 1% alginate hydrogel and the NPs for the three concentrations tested (Figure 9). Meanwhile, for the hydrogel in the flow direction, 2.6%, 10.6%, and 15.4% of the initial mass loadings (0.08 µg, 0.16 µg, and 0.33 µg) diffused in the Alg:M hydrogel, whereas almost nothing diffused in Alg:M against the flow. Interestingly, at a 3-μL/min flow rate, 26.2%, 38.8%, and 43.1% of the initial mass loadings of 0.08 µg, 0.16 µg, and 0.33 µg, respectively, diffused through Alg:M in the flow direction, whereas 6.6%, 8.2%, and 14.4% were found in Alg:M against the flow direction (Figure 9). In addition, low percentages of the molecule remained trapped in 1% alginate hydrogel containing the NPs. However, the use of a 6.5-μL/min flow rate allowed a cumulative percentage release >75% for the three mass loadings, suggesting that the diffused percentages of CXCL-AF647 in the Alg:M hydrogel were minor. Indeed, only 2.3%, 7.1%, and 12.4% of the initial mass loadings diffused in the Alg:M that followed the flow direction. Additionally, since ∼90% of the initial mass loadings of the CXCL12-AF647 was released under 10 μL/min flow rate, 0.9%, 1.4%, and 8.1% of 0.08 µg, 0.16 µg, and 0.33 µg, respectively, diffused in the Alg:M hydrogel in the flow direction, whereas less than 1% of 0.33 µg diffused in the Alg:M hydrogel against the flow.

#### 3.3.2 Fluorescence scans

The hydrogel scans were assessed using the EVOS^®^ FL Auto Imaging System (Life Technologies) to follow the fluorescence of CXCL12-AF647—hence, its distribution over time. The NPs allowed the observation of the CXCL12-AF647 through fluorescence since it concentrates in single points, unlike when it is free and uniformly dispersed in the hydrogel (free CXCL12-AF647 condition). Scans were in accordance with the molecule quantification (Figure 9). The scans were taken at t = 0, t = 24 h, and at the end of every experiment determined when no more CXCL12-AF647 was detected for several consecutive time points. At t = 0, the fluorescence of CXCL12-AF647 in all the experiments increased when the initial mass loading increased. We also observed that the fluorescence was concentrated in the middle of the gels, as expected. As the flow rate increased, the fluorescence decreased in time.

**FIGURE 10:**
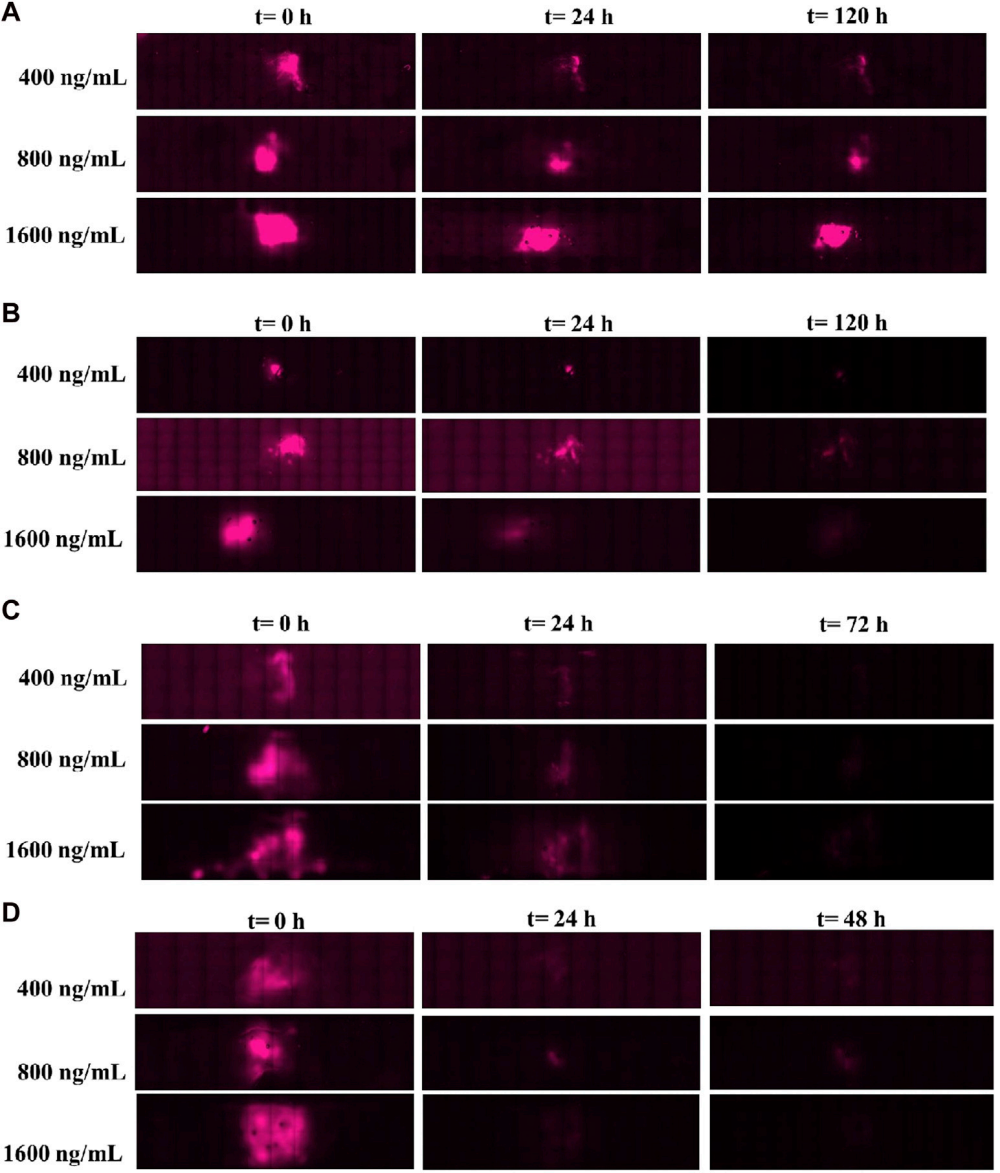
Images of the culture chambers during the kinetic release experiment for the different mass loadings of Alg/Chit-NPs-CXCL12-AF647 under different flow rates: **(A)** 0.5 μL/min and **(B)** 3 μL/min, **(C)** 6.5 μL/min, and **(D)** 10 μL/min.

After 120 h of release kinetics under a 0.5-μL/min flow rate (Figure 10A), fluorescence was still observed for the three concentrations, meaning that most of the CXCL12-AF647 mass loadings were not released and remained trapped in the hydrogel. For the same period, using a higher flow rate (3 μL/min), the fluorescence signal was less intense (Figure 10B). This suggests that a certain amount of the initial mass was released, whereas the other amount may be diffused in the Alg:M (50:50) or remained trapped in 1% alginate hydrogel. When the experiment was run at 6.5 μL/min (Figure 10C), the fluorescence disappeared in less than 48 h, suggesting that the different quantities loaded were released quickly from the alginate hydrogel. The same behavior of CXCL12-AF647 was observed using a higher flow rate (10 μL/min), except that the fluorescence disappeared in 24 h (Figure 10D). These observations are in accordance with the quantification of the CXCL12-AF647 release and the evaluation of its distribution in the various hydrogel/culture media.

## 4 Discussion

The efficiency of standard treatments for GBM is very limited, mostly because of the inability to target GBM cells that migrate out of the tumor to the surrounding brain parenchyma. Regulators such as the CXCL12–CXCR4 axis, the tumor microenvironment, and the IFF are known to be important for GBM cell migration (Radisky et al., 2002; Do Carmo et al., 2010; Munson et al., 2013). To develop future GBM treatments without affecting healthy brain tissue, the design of models that mimic the different phenomena that occur in the brain is crucial. There seem to be no 3D *in vitro* models that mimic the human brain cerebral fluid flow in the research of GBM treatment. In the present study, we developed a model that recapitulates the peritumoral environment that mimics the IFF to study CXCL12 release under conditions closer to the human brain’s environment.

In our previous work, we found that Alg/Chit-NPs were adapted to encapsulate a positively charged protein CXCL12 at physiological pH with an encapsulation percentage higher than 96.7% and an average size of approximately 250 nm (Gascon et al., 2020). In addition to the NP’s size, zeta potential is another important parameter in several phenomena, including drug delivery which affects the NP’s stability (Patel and Agrawal, 2011; Honary and Zahir, 2013; Griesser et al., 2017). Generally, the zeta potential of the particles is affected by the pH of the solution in which the NPs are dispersed, the ionic strength, and the ions present in the particle suspension (Saupe et al., 2006). A zeta potential value higher than +30 mV or less than –30 mV indicates good stability of the particles by blocking the contact between them caused by electric repulsion, consequently reducing aggregation (Saupe et al., 2006). In the present study, we found that the zeta potential of Alg/Chit-NPs with or without CXCL12-AF647 has a positive charge (41.73 mV and 40.03 mV, respectively) (Figures 5E, F), which may be attributed to the positively charged chitosan present on the particles’ surface. This indicates that the repulsive forces between the NPs are high, reducing the risk of aggregation. Furthermore, the encapsulation of a positively charged molecule such as CXCL12 does not seem to significantly affect the stability of the Alg/Chit-NPs as previously reported (Costa et al., 2015). Similarly, Katuwavila et al. (2016) encapsulated doxorubicin (DOX) in Alg/Chit-NPs to maintain its delivery in the blood stream as an anticancer agent. The zeta potential values of Alg/Chit-NPs with and without DOX were 36 mV and 35 mV, respectively, proving that the encapsulation of DOX does not affect Alg/Chit-NP stability (Katuwavila et al., 2016).

Alginate is structurally similar to hyaluronic acid, a primary component of brain ECM (Moxon et al., 2019), so it is a biomaterial that has been widely used in 3D culture systems (Moxon et al., 2019; Nii et al., 2020). Alginate-gelatin hydrogel replicates the mechanical behavior of soft tissues such as the brain tissue, suggesting that alginate is a very promising candidate for tissue engineering applications (Distler et al., 2020). In addition, Cavo et al. (2018) reported that alginate:Matrigel at a 50:50 mixing ratio allowed human breast cancer cells (MDA-MB-231) to migrate by replicating the 3D tumoral microenvironment. This hydrogel promotes breast cancer cell invasion while allowing these cells to express their malignant characteristics (Cavo et al., 2018). Consequently, we studied the release of CXCL12-AF647 from 1% alginate hydrogel. We then investigated the release kinetics of CXCL12-AF647 from Alg:M (50:50) hydrogel as it imitates the peritumoral space (Cavo et al., 2018). The cumulative proportions of CXCL12 released from 1% alginate hydrogel were not influenced by the initial mass loading compared to when they were released from the Alg:M (50:50) hydrogel. For all the experimental conditions, we observed an initial burst release in the first 3 h that was followed by a sustained release, with a pseudo-plateau reached after 72 h (Figure 6). This burst release may be explained by some remaining unbounded CXCL12 molecules which did not interact with the negative alginate chains. The main mechanism guiding CXCL12 release from both hydrogels was diffusion, as determined by the Korsmeyer–Peppas model (Korsmeyer et al., 1983). The exponential parameter decreased from 0.45 to 0.29 as the initial mass loading increased, indicating the presence of strong electrostatic interactions (Lauzon et al., 2014). The presence of interactions between the protein and the scaffold carrying it has been reported in different studies (Koutsopoulos et al., 2009; Lauzon et al., 2018; Gascon et al., 2020). In addition, D_eff_ values of ∼1.5 × 10ˉ⁹ $m^{2}\times$ $s^{-1}$ for CXCL12 release regardless of the initial mass loading were obtained, which is normally expected for such a small protein (Table 2) (Koutsopoulos et al., 2009). The small variations in diffusion coefficient values in respect to initial mass loading were also coherent with our hypothesis of a constant diffusion coefficient in Fick’s second law-based mathematical framework (Hecq et al., 2015; Lauzon et al., 2018; Gascon et al., 2020). Our results accord with those of Koutsopoulos et al. (2009), who studied the release of different proteins through nanofiber Ac-(RADA)4-CONH2 peptide hydrogels. They proved that the protein’s diffusion depends primarily on its size and charge (Koutsopoulos et al., 2009). For the release of a small protein (∼14 kDa, pI = 11.4) inside the hydrogel, a burst release was observed in the first 8 h followed by a sustained release. The burst release is most likely due to molecules near the solvent–hydrogel interface which allowed them to diffuse rapidly in the solution. Furthermore, they found a diffusion coefficient of ∼0.5 × 10ˉ^1^⁰ $m^{2}\times s^{-1}$ compared to 0.96 × 10ˉ^1^⁰ $m^{2}\times s^{-1}$ in the solution (Koutsopoulos et al., 2009). A solution diffusivity value of ∼1.04 × 10ˉ^1^⁰ $m^{2}\times s^{-1}$ is normally expected with such a protein, which was explained by the presence of interactions between it and the nanofiber hydrogel (Koutsopoulos et al., 2009). CXCL12 diffusion coefficient values (Table 2) from Alg:M hydrogel were in the same order of magnitude as reported by Branco et al. (2010) for a small protein (α-lactalbumin) of 14.1 kDa (4.2–4.5 pI) (40 × 10ˉ⁸ ${cm}^{2}\times s^{-1}$ ). In contrast, as the initial mass loading increases, we observed a decrease of the overall mass transfer coefficient, indicating the presence of strong electrostatic interactions of CXCL12 with the different elements present in the Matrigel. Matrigel is extracted from the Engelbreth–Holm–Swarm mouse sarcoma and is rich in extracellular matrix proteins such as laminin, collagen IV, proteoglycans, and growth factors [43]. These components may interact with the CXCL12 and form aggregates as the initial mass increases.

Our study also showed that the release of CXCL12 can be tuned for the three initial mass loadings by encapsulating it in Alg/Chit-NPs and embedding in 1% alginate (w/v) hydrogel for better control of the molecule release. Lower cumulative percentages of release and a stronger attenuation of the burst release than the unloaded CXCL12 were observed, implying the presence of resistance at the interfaces between the NPs and the surrounding hydrogel (Figures 7A–C). This can be potentially explained by the two steps in which CXCL12-AF647 is released: i) CXCL12-AF647 diffuses from the NPs to alginate hydrogel as observed in the first hours of the release profile; ii) the released molecules in the hydrogel interact with the negatively charged alginate chains, which could cause them to be trapped, with a consequent decrease in the release of CXCL12-AF647 in the culture media. Similarly, Molina-Peña et al. (2021) loaded CXCL12 in polylactic-co-glycolic acid (PLGA) and PEG-PLGA NPs and embedded the system into a nanofibrous scaffold of chitosan solution with fiber-forming additive polyethylene oxide. The use of the nanofibrous scaffolds with the NPs allowed a sustained release for up to 35 days, compared to 5 days when the molecule was unencapsulated, and an attenuation of the burst release (Molina-Peña et al., 2021). The sustained release was also explained by a two-step process in which CXCL12 diffuses first from PEG-PLGA NPs into the nanofibers before it can be released into the culture media (Molina-Peña et al., 2021). In the present study, mathematical modeling allowed us to determine the D_eff_, which was an order of magnitude lower than the free CXCL12. Lower diffusion coefficients were obtained for the three experimental conditions (Table 3), which could be also explained by the two-step release mechanism of CXCL12. Consequently, the molecule needed more time to diffuse, which was in accordance with the release profile obtained. In the same framework, Yu et al. designed anionic liposomes loaded with CXCL12 (lipo-CXCL12) and embedded them in gelatin methacrylate (GelMA) to form a nanocomposite hydrogel (Justine et al., 2020). They found that the diffusion coefficient of lipo-CXCL12 (2 × 10ˉ⁸ $cm^{2}\times s^{-1}$ ) released from liposomes was two orders of magnitude lower than free CXCL12, which leads to a slower diffusion in an aqueous environment. This agrees with their release kinetics study in which the positively charged CXCL12 was slowly released due to the charge–charge interactions with the negative charge of type B-derived GelMA. This may explain why the CXCL12 remained trapped in the GelMA fibers and consequently slowed the release from the hydrogel.

We then developed a dynamic *in vitro* 3D model using a perfusion bioreactor to mimic the IFF for better comprehension of CXCL12 release under physiological-like conditions (Figure 2). This unique model allowed us to track the release of CXCL12 in time and space inside the 3D hydrogels. We used four flow rates to recapitulate the different zones in which GBM tumors may be located (Zhu et al., 2006; Kingsmore et al., 2016; Cornelison et al., 2018). Our study showed that, independent of the initial mass loading, the cumulative CXCL12-AF647 proportions released increase when the flow rate increases (Figure 10). Less than 7.3% of the mass loadings was released with a low flow rate of 0.5 μL/min, which is lower than that observed in static conditions. However, to promote GBM cell chemotaxis, CXCL12 at a concentration of 100 ng/mL is required, which was not reached at such a flow rate. This suggests that if a GBM tumor is in a zone in which IFF is ∼ 0.5 μL/min, the initial CXCL12 dose loaded should be increased. Results indicate that CXCL12-AF647 may remain trapped in the hydrogel or diffused through the Alg:M (50:50). To verify those assumptions, each section of the gels was dissociated to quantify the remaining CXL12-AF647 at the end of the experiment. More than 75% of the initial mass loadings were contained in the 1% alginate hydrogel and the NPs, whereas low percentages diffused in Alg:M (50:50) hydrogel in the flow direction (Figure 9). The release kinetics study was confirmed with scans in which the molecule fluorescence was still observed after 120 h at the end of each experiment. When the flow rate was increased to 3 μL/min, higher CXCL12-AF647 proportions were released, suggesting that the contribution of the convective flow impacted the chemokine release and distribution within the gel. For the three experimental conditions, a high proportion of the chemokine diffused through the Alg:M in and against the flow direction, which also accorded with the fluorescence scans. Consequently, we assume that both diffusion and convection phenomena are driving the movement of the CXCL12 within the system (Figure 10). At high flow rates, only a small amount of the molecule remained trapped, and the fluorescence disappeared in less than 48 h, suggesting that convection is the dominant mechanism. There was also a sharp cutoff in terms of chemokine release out of the source (central alginate hydrogel section) and the gels, as most of the drug was collected in the culture medium. From our observation, it appears that there is a defined flow range that promotes the distribution of the chemokine in the simulated brain parenchyma. In relation to future work, those results are very useful for creating a concentration gradient for GBM cell attraction (100 ng/mL) by chemotaxis and determining the chemokine dose required to promote and direct cancer cell migration in respect to the location in the brain (low vs. highly irrigated zone).

Different studies highlighted the impact of dynamic culture conditions upon cell migration but not in the context of drug kinetic releases. For example, Munson et al. (2013) found interstitial flow effect to be an active regulator of GBM cell invasion. Their finding showed that GBM cell invasion is enhanced by CXCL12 gradient (100 nmol/L). Furthermore, dynamic culture conditions that mimic the fluid flow (velocity of 0.7 μm/s) enhanced GBM cell migration via the CXCL12-CXCR4 signaling mechanism (Munson et al., 2013). These results suggest IFF as a modulator of GBM cell spreading because it can also stimulate those cells for CXCL12 secretion (Munson et al., 2013). Furthermore, Cavo et al. (2018) demonstrated with a perfusion bioreactor that breast cancer cells were able to migrate through an Alg:M (50:50) hydrogel under flow. While their system lacked control over the flow rate, which is an important parameter that may influence cell migration, these studies highlight the importance of designing dynamic *in vitro* 3D models to better understand cancer cell migration behaviors. In the future, we intend to use this new 3D *in vitro* model to incorporate Alg/Chit-NPs loaded with the chemokine CXCL12 in a biocompatible polysaccharide-based macroporous hydrogel matrix to attract GBM cells. Through this future work, we also aim to assess GBM cell behaviors in response to the resulting chemokine gradient subjected to various flow while allowing them to express their invasive characteristics in a 3D environment.

## 5 Conclusion

In the present study, we assessed the release kinetics of CXCL12-AF647 from alginate-based hydrogel as a potential future therapeutic approach against GBM in both static and dynamic culture conditions. The release of free CXCL12-AF647 was primarily diffusive under static conditions, and the use of Alg/Chit-NPs allows better control and sustained release of the molecule over time. CXCL12 released under dynamic conditions was evaluated using a unique *in vitro* 3D model that simulates IFF found in the brain. The use of different flow rates allowed an understanding of the contribution of the brain parenchyma fluidic phase on the molecule kinetic release and distribution in respect to time and space. The impact of low flow rates that reflect poorly irrigated brain zones had a slow and diffusion-driven release compared to high flow rates that mimic well-irrigated zones in which convection was predominant. This specific result is of great interest as it can help fine-tune the chemokines initial mass required to create the desired gradient in a clinical context. We intend in upcoming work to assess the capacity of our system to promote GBM cell attraction under CXCL12 gradient in 3D dynamic culture conditions; this will allow us to understand their behavior in an environment that mimics the brain parenchyma. Since the convective contribution of the brain fluid remains poorly understood, the 3D model developed in this study will also contribute to the development of other therapeutic strategies targeting not only brain cancer but also brain degenerative disorders such as Parkinson’s and Alzheimer’s diseases.

## Data Availability

The raw data supporting the conclusion of this article will be made available by the authors, without undue reservation.
